# Benzyl isothiocyanate ameliorates cognitive function in mice of chronic temporal lobe epilepsy

**DOI:** 10.3389/fneur.2024.1330102

**Published:** 2024-04-23

**Authors:** Chang Xiaoyu, Zhou Hongzhen, Peng Nan, Gao Tengwei, Gong Yanan, Guo Yan, La Haiyan, Ma Li, Wu Haiya, Wen Yujun, Zhang Rui

**Affiliations:** ^1^School of Public Health and Management, Ningxia Medical University, Yinchuan, China; ^2^Editorial Board of Journal of Ningxia Medical University, Yinchuan, China; ^3^Ningxia Key Laboratory of Cerebrocranial Disease, Incubation Base of National Key Laboratory, Ningxia Medical University, Yinchuan, China; ^4^Key Laboratory of Environmental Factors and Chronic Disease Control, Ningxia Medical University, Yinchuan, China

**Keywords:** temporal lobe epilepsy, benzyl isothiocyanate, neuroprotective effect, cognitive function, Nrf2

## Abstract

**Objective:**

Temporal lobe epilepsy (TLE) is a prevalent refractory partial epilepsy seen in clinical practice, with most cases originating from the hippocampus and being characterized by impaired learning and memory. Oxidative stress plays a direct role in the development of epilepsy and neurodegeneration while promoting cognitive dysfunction. Previous research indicates that benzyl isothiocyanate (BITC) has antioxidative stress properties and contributes to neuroprotection. In this study, we aimed to investigate the neuroprotective effect of BITC on a lithium-pilocarpine-induced temporal lobe epileptic mice model.

**Methods:**

We conducted Intellicage learning tests, Morris water maze, open field test, and step-down-type passive avoidance tests, respectively. In addition, body weight and brain-to-body ratio were calculated. Nissl staining, real-time quantitative PCR detection of nuclear factor erythroid 2-related factor 2 (Nrf2), heme oxygenase 1 (HO-1) and NAD(P)H dehydrogenase quinone 1(NQO1) were performed. Content of malondialdehyde (MDA) and activities of superoxide dismutase (SOD), glutathione peroxidase (GSH-Px) and total antioxidant capacity (T-AOC) were determined.

**Results:**

Our results demonstrate that BITC enhances cognitive function and motor ability in mice, as determined by Intellicage learning tests, Morris water maze, open field test, and step-down-type passive avoidance tests, respectively. Epilepsy leads to the loss of neurons in the CA3 region, while BITC treatment plays a positive role in neuroprotection, especially in the cortex. In comparison to the control group, the EP group exhibited decreased transcription levels of HO-1 and NQO1, alongside reduced GSH-Px activity, while MDA content was elevated. Conversely, the BITC treatment group, when compared to the EP group, showed enhanced transcription levels of Nrf2, HO-1, and NQO1, along with increased GSH-Px activity, and a decrease in MDA content.

**Conclusion:**

In summary, our study provides evidence that BITC can improve cognitive impairments in pilocarpine-induced epileptic mice, demonstrating significant antioxidant effects and neuroprotective properties. This highlights its potential as a phytochemical for managing the sequelae of epilepsy.

## 1 Introduction

Epilepsy is a clinical syndrome characterized by highly synchronized abnormal neuronal discharges in the brain, resulting from various etiologies (1). It is a common neurological disorder, with approximately 30% of cases being refractory epilepsies (2). Among these, temporal lobe epilepsy is the most prevalent type (3). Patients with temporal lobe epilepsy often exhibit a wide range of cognitive dysfunctions, which significantly impairs their quality of life (4, 5). Good cognitive function is essential for daily activities that require perception, attention, memory, and other cognitive processes (6). However, temporal lobe epilepsy can cause structural abnormalities in multiple cognitive brain areas, slow down cognition, diffuse functional connections, and severely impair patients’ cognitive function (7). Additionally, the increase in oxidative stress persists throughout the development of epilepsy, leading to neuronal damage and cognitive impairment (8). Intellicage learning tests, Morris water maze tests, open field tests, and step-down type passive avoidance tests are widely used to evaluate rodents’ learning and memory abilities (9). Nerve cells are the most fundamental structural and functional units of the nervous system (10), and the number of nerve cells closely relates to cognitive function and memory ability. The hippocampal region is a vital component of the limbic system, which regulates learning, memory, and behavior (11). Therefore, the number of nerve cells and the results of neurobehavioral tests can reflect the neurodegenerative changes in animals to some extent. While surgery is the preferred method for the treatment of refractory temporal lobe epilepsy, it carries an elevated risk, a high cost, and many factors that affect the outcome (3). Therefore, there is an ongoing need for research and application of economic, safe, and effective antiepileptic drugs in temporal lobe epilepsy.

In recent years, the bioactive substances in cruciferous vegetables have attracted great attention in the field of health and wellness. Phenolic compounds, organic sulfides, carotenoids, and vitamins collectively contribute to the health-promoting attributes of cruciferous vegetables (12). Among these, benzyl isothiocyanate (BITC) is a natural component with remarkable properties. Numerous studies have demonstrated that BITC has a variety of beneficial effects, including anti-inflammatory, antioxidant, anti-cancer, and cytoprotective properties (13–15). BITC has been the subject of several studies due to its potential in modulating the levels of malondialdehyde (MDA), catalase (CAT), superoxide dismutase (SOD), and glutathione peroxidase (GSH-Px), and enhancing the cellular antioxidant defense mechanism (16). The nuclear factor erythroid 2-related factor 2 (Nrf2) pathway plays a vital role in the regulation of antioxidant enzymes (17). Moreover, the ability of BITC to modulate inflammation, oxidative stress, and apoptosis through Nrf2/ heme oxygenase 1 (HO-1) and nuclear factor kappa-B (NF-κB) signaling pathways has been highlighted (13). It has been suggested that some plant-derived phytochemicals, including BITC, could be effective in the prevention and/or management of diseases associated with oxidative stress and inflammation (18). Despite the insights provided by previous studies regarding the molecular role of BITC, there are still significant gaps, particularly in understanding its potential impact on neurological function, especially in pilocarpine-induced epileptic mice. The complex interaction between bioactive compounds and neurological function remains a compelling area of inquiry. Consequently, this study aims to bridge this knowledge gap by investigating whether BITC can indeed improve the cognitive impairment in epileptic mice and play a neuroprotective role. By exploring this unknown aspect, our objective is to contribute valuable insights to the ongoing discussions on the multifaceted benefits of cruciferous vegetables and their potential applications in promoting neurohealth. This investigation is anticipated not only to enhance our comprehension of the impact of BITC, but also to pave the way for new therapeutic interventions in the field of cognitive health.

## 2 Materials and methods

### 2.1 Reagent

BITC (purity: ≥ 98.0%), pilocarpine (purity: ≥ 98%), and atropine (purity: ≥ 98.0%) were obtained from Sigma-Aldrich Corporation (St Louis, Missouri); lithium chloride was purchased from Tianjin Haijing Chemical Co. LTD (Tianjin, China); Nissl staining solution was obtained from Beyotime Corporation (Shanghai, China); total RNA extraction kit (Omega), reverse transcription kit, RT-PCR SYBR kit (Dalian Takara); GSH-Px, T-SOD, T-AOC, and MDA assay kits (Nanjing Jiancheng Bioengineering Institute). All other reagents were obtained from commercial sources with the highest purity available.

### 2.2 Animals and treatment

Healthy 20-week-old C57BL/6J mice were provided by Ningxia Medical University Experimental Animal Center (IACUC Animal code: SCXK (Ning) 2017-0001). Mice were kept in a 12-h cycle of light and dark, with temperatures controlled at 22°C ± 2°C, the humidity of 55% ± 15%, and food and water *ad libitum*. The Animal Care and Use Committee of Ningxia Medical University approved the experiment (no. NXMU2020-021), which complied with the National Institute of Health’s Guide for the Care and Use of Laboratory Animals. All efforts were made to minimize suffering and the number of animals.

An acute epilepsy model in mice was established by randomly dividing male mice into three groups (11 per group): a control group, an epilepsy (EP) group, and an EP group treated with BITC (EP + BITC). Mice in the EP + BITC group were administered 10 mg/kg BITC dissolved in corn oil via gavage once daily; mice in the EP and control groups were injected with an equivalent volume of corn oil as a placebo, with the treatment lasting for 7 days. The temporal lobe epilepsy model in mice was induced as follows: first, lithium chloride (130 mg/kg body weight) was injected 18 h before the administration of pilocarpine (290 mg/kg body weight). Then, 2 h later, anesthesia was performed using isoflurane, and the brain was extracted. In each group, 3 mice were selected for perfusion to collect brain tissue, and the remaining 8 were used to obtain fresh brain tissue samples.

**Figure 1:**
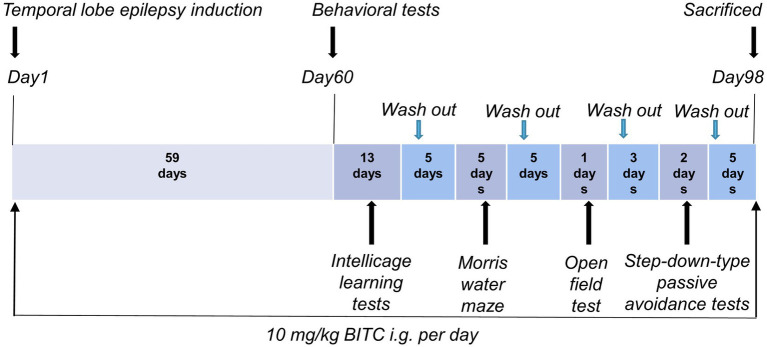
Scheme of animal treatments.

Status epileptic mouse model: Male mice were randomly divided into 3 groups (*n* = 10) by weight: a control group and epilepsy (EP) group with or without BITC treatment. Animal treatments are shown in timeline (Figure 1). Temporal lobe epilepsy was induced in epilepsy group and BITC intervention group (EP + BITC) at the beginning of the experiment whereas the mice in control group were treated with saline. The lithium-pilocarpine-induced temporal lobe epileptic mice was carried out as follows: Mice were injected with lithium chloride (130 mg/kg bodyweight, i.p.) 18 h before pilocarpine (290 mg/kg bodyweight, i.p.) injection. Mice in the EP + BITC group were treatment by gavage with 10 mg/kg BITC (dissolved in corn oil) once a day during the whole experiment, and mice in the EP group and control group were injected with gastric equivalent vehicle. All mice were weighed weekly.

Behavioral tests for mice were performed on the 60th days after treatment, and then animals were sacrificed 5 days after the last behavioral test to calculate brain-to-body weight ratio and to conduct Nissl’s Staining.

### 2.3 Behavioral tests

The behavioral test for mice was performed in a quiet enclosed room at a temperature of 22 ± 2°C. There is a video camera connected to a computer directly above the center of the experimental area during Morris water maze, open field test, step-down-type passive avoidance tests.

#### 2.3.1 Intellicage learning tests

Intellicage (TSE Systems GmbH, Germany) is equipped with four chambers located in four corners. Each chamber contains two water bottles and a transponder reader antenna that registers the microchip of the entering mice. Infrared beam-break sensors were programmed to keep open or closed upon nose-poke response. Microchips were implanted into subcutaneous part of nuchal region of each mouse to ensure data to be separated by each individual mouse. The experimental procedure was as follows [refer to previously described with major modification (19)]:

Divided into 5 parts:

Free exploration: The mice were allowed to explore freely and familiarize themselves with the environment for 3 days. All doors were opened, and all water bottles were available. The number of corner visits and numbers of drinks of each mouse were recorded.

Nose-poke learning: During these 3 days, all doors were closed initially, and mice need to nose-poke to open the door to drink water. The number of corner visits were recorded to calculate corner preference of each mouse.

Behavioral extinguishment: Mice were allowed to do free exploration for 1 day to extinguish the previous learning behavior.

Positive position learning: During 3 days of test, each mouse’s least preferred corner was defined as “right” corner, while the remaining corners would be defined as “wrong” corners. The doors would be opened and the mice were allowed to drink after they nose-poked the “right” corner. The number of right visits and number of drinking were recorded to evaluate the positive position learning abilities of the mice.

Reversed position learning: During these 3 days, the opposite corner of the previous “right” corner would be defined as the new “right” corner, while the remaining corner would be defined as new “wrong” corners. The doors would be opened and the mice were allowed to drink after they nose-poked the “right” corner. The number of right visits and number of drinking were recorded to evaluate the reversed position learning abilities of the mice.

All mice were washed out for 5 days at the end of Intellicage learning tests.

#### 2.3.2 Morris water maze

A five-day Morris water maze procedure was carried out as described previously (20) after Intellicage learning tests, and the escape latency in escape trials and the passing times in the probe trials were recorded. And the mice were washed out for 5 days at the end of Morris water maze.

#### 2.3.3 Open field test

The test was executed as described previously (20) after Morris water maze, and immobility time in the central area (latency), times of cross grids, and times of uprights were recorded. A three-day wash-out period would be conducted after the open field test.

#### 2.3.4 Step-down-type passive avoidance tests

YLS-3TB platform recorder (Yiyan Technology, Jinan, China) was used to conduct Step-Down-Type passive avoidance tests follow the methods we described before (21). Escape latency and numbers of errors in training session and Step-down latency and numbers of errors in retention test were recorded.

The mice would be sacrificed after a five-day wash-out.

### 2.4 Brain-to-body weight ratio

After anesthetized with isoflurane, the mice were weighed, and brains were quickly isolated and washed with ice-cold saline. The brain-to-body weight ratio were calculated according to the following formula:

$\text{Brain}-to-\text{body}\;\text{weight}\;\text{ratio}=\text{Brain}\;\text{mass}\;\left(\text{mg}\right)/\text{Weight}\;\left(g\right)$

### 2.5 Nissl’s staining

The brains from Bregma −2.5 mm to −3.5 mm were removed, post-fixed with 4% paraformaldehyde (pH 7.4), and dehydrated with 30% sucrose solution. Coronal sections (40 μm) were obtained with a cryostat and washed with phosphate buffer. After stained with crystal violet solution at 56°C for 30 min, followed by washing with distilled water. Sections were differentiated with 1% hydrochloric acid ethanol solution until the backgrounds were nearly colorless. Dehydration was carried out by 70% alcohol, 80% alcohol, and 95% alcohol, respectively, for 2 min, and then by anhydrous alcohol twice for 5 min. Then sections were transparentized with xylene, and sealed. The changes in the morphology and number of nerve cells in hippocampal CA1, CA3, and CORTEX areas were observed with an optical microscope.

### 2.6 Real-time quantitative PCR

Real-time quantitative PCR was employed to assess the mRNA levels of HO-1, NAD(P)H dehydrogenase quinone 1 (NQO1), and Nrf2 in the hippocampal tissues of diverse mouse groups. Initially, total RNA was extracted from each sample, and the total mRNA concentration was standardized to 500 ng/μL. Subsequently, mRNA underwent reverse transcription to form cDNA. The reverse transcription reaction system comprised: 5X PrimeScript RT Master Mix (Perfect Real Time) 2 μL, total mRNA 1 μL, and sterile water 7 μL.

GAPDH served as the internal reference, followed by PCR amplification. The specific primers were as follows:

HO-1 primers:

Forward: 5’-CAAGCCGAGAATGCTGAGTTCATG-3’

Reverse: 5’-GCAAGGGATGATTTCCTGCCAG-3’

NQO1 primers:

Forward: 5’-AGGATGGGAGGTACTCGAATC-3’

Reverse: 5’-AGGCGTCCTTCCTTATATGCTA-3’

Nrf2 primers:

Forward: 5’-AAAATCATTAACCTCCCTGTTGAT-3’

Reverse: 5’-CGGCGACTTTATTCTTACCTCTC-3’

The PCR amplification system comprised: TB Green Premix Ex Tap II 12.5 μL, forward primer 1 μL, reverse primer 1 μL, Sterile water 8.5 μL and cDNA 2 μL. Reaction conditions were as follows: pre-denaturation at 95°C for 30 s, followed by 40 cycles, each cycle involving denaturation at 95°C for 15 s, annealing at 58°C for 30 s, and extension at 72°C for 30 s.

### 2.7 Enzymatic assay measurements

Isolate the mouse cortex and use an ultrasonic tissue disruptor to prepare a 10% tissue homogenate, centrifuge at 2000 rpm for 15 min at 4°C, and carefully aspirate the supernatant. The activity of tissue T-SOD, MDA, GSH-Px, and total antioxidant capacity (T-AOC) were determined and calculated as follows. Protein concentration: Take the sample to be tested, add Coomassie Brilliant Blue G-250 solution for color development, mix thoroughly, then use an enzyme-linked immunosorbent assay reader at 595 nm wavelength for colorimetry, record the absorbance value and calculate the protein concentration in the solution based on the standard curve. T-SOD: Dilute the above tissue homogenate to 2.5%, add substrate and incubate at 37°C for 20 min, read with an ELISA reader at 450 nm, and calculate. SOD activity = (OD value after enzyme tube zeroing - OD value after non-enzyme tube zeroing) × 1,000 / (OD value after enzyme tube zeroing × 134 × 2.5% tissue homogenate protein concentration). MDA: Use a 10% tissue homogenate to prepare the reaction system, water bath at 95°C for 40 min, centrifuge at 3800 rpm for 10 min, read with an ELISA reader at 532 nm, and calculate. MDA content = [(OD value after zeroing – OD value after blank zeroing) × 10 / (OD value after standard zeroing – OD value after blank zeroing) × 10% tissue homogenate protein concentration × 10]. GSH-Px: Prepare a 2.5% tissue homogenate, color development after enzymatic reaction for 15 min, read with an ELISA reader at 412 nm, and calculate. GSH-Px activity = (OD value after non-enzyme tube zeroing – OD value after enzyme tube zeroing) × 5 / ((standard zeroing – blank zeroing) × 5 × 0.2 × 0.25% tissue homogenate protein concentration). T-AOC: Use a 10% tissue homogenate to prepare the reaction system, react at room temperature for 10 min, read with an ELISA reader at 520 nm, and calculate. T-AOC = (OD value – concentration after blank zeroing) × 10 / ((concentration after standard zeroing – concentration after blank zeroing) × 10% tissue homogenate protein concentration).

### 2.8 Statistical analyses

Parametric data were presented as means ± standard error of the means (Mean ± SEM). Data were plotted using GraphPad 9.0 software. If the data were normally distributed and had equal variance, one-way ANOVA was used for analysis, and LSD-t test was used for multiple comparisons between groups. Data that were not normally distributed were analyzed using the Kruskal-Wallis H test. *α* = 0.05, *p* < 0.05 was considered statistically significant.

## 3 Results

### 3.1 Effect of BITC on epileptic mouse model and body weights

**Figure 2:**
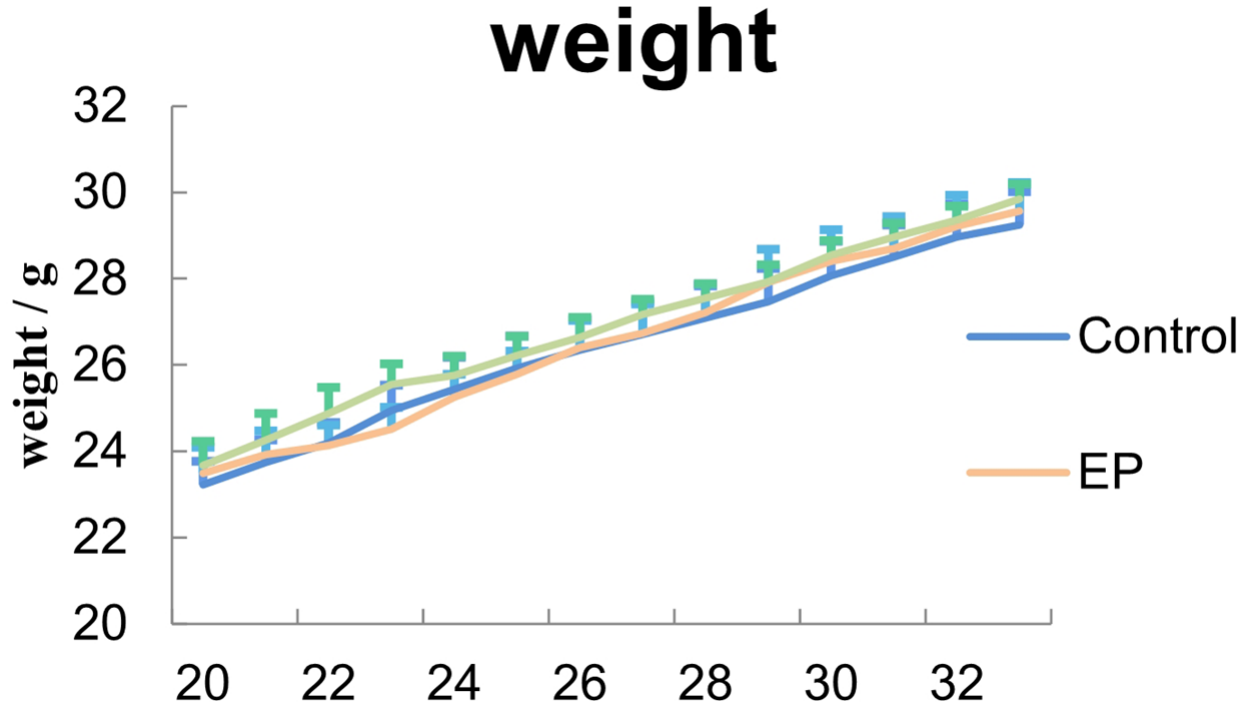
Measurement of mice body weight. During the treatment, no significant difference in body wights was observed between control, EP and EP + BITC mice (*n* = 10; mean ± S.E.M.; One-way ANOVA followed by *post hoc* least significant difference [LSD] multiple comparison tests).

During the acute phase of epilepsy, there was no significant difference in the latency of seizure onset between the groups of mice (*p* > 0.05) (Supplementary Figure S1D). Compared to the control group, the average power of δ waves increased in the EP group and EP + BITC group (*p* < 0.05); there was no significant difference in the average power of δ waves between the EP group and EP + BITC group (*p* > 0.05) (Supplementary Figure S1E). During the treatment period, no sign of significant toxicity was observed in all mice, and there were no significant differences (*p* > 0.05) in body weight of mice between groups for each week (Figure 2). The results indicate that the BITC treatment did not impact the latency of seizure onset or the average power of δ waves in mice. Additionally, the BITC treatment did not cause significant physiological alterations or modifications to the growth pattern of mice.

### 3.2 Intellicage learning tests

**Figure 3:**
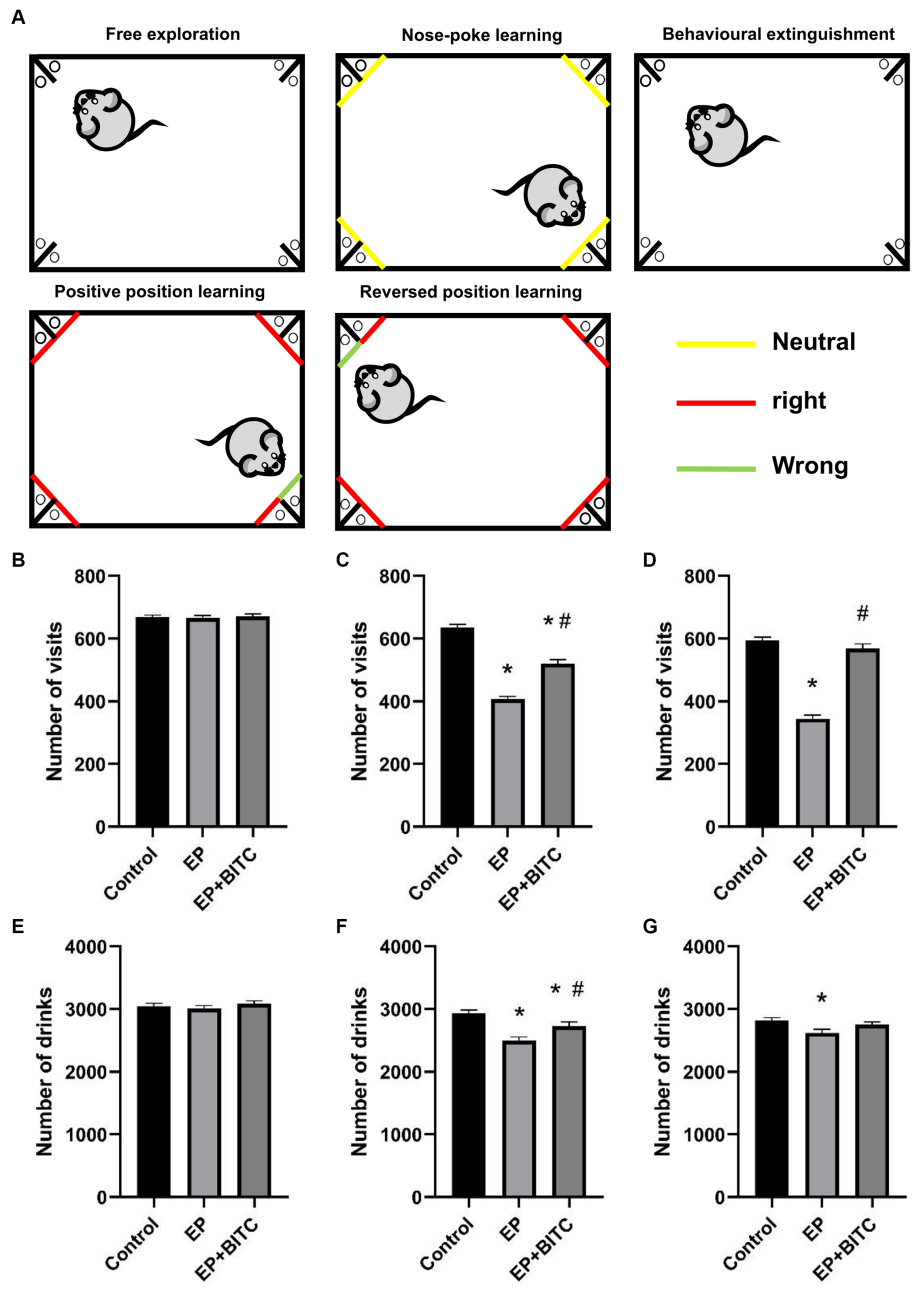
Intellicage. Analysis of Intellicage test in mice of control, EP, and EP + BITC (*n* = 10 per group; mean ± S.E.M.). **(A)** Intellicage learning module design. **(B)** The number of visits of three groups of mice in free exploration. No difference between the control, EP, and EP + BITC groups, *p* > 0.05. **(C)** The number of drinks of three groups of mice in free exploration. No difference in weight gain between the control, EP, and EP + BITC groups, *p* > 0.05. **(D)** The number of visits of three groups of mice in position learning. **(E)** The number of drinks of three groups of mice in position learning. **(F)** The number of visits of three groups of mice in reversal position learning. **(G)** The number of drinks of three groups of mice in reversal position learning. * Compared with control group: *p* < 0.05, # compared with EP group: *p* < 0.05. One-way ANOVA followed by *post hoc* least significant difference [LSD] multiple comparison tests.

As shown in Figure 3, at free exploration part, there was no difference in terms of the number of visits and number of drinks between groups (*p* > 0.05). During positive position learning, mice in EP group with or without BITC treatment exhibited a smaller number of visits and drinks compared with mice in the control group (*p* < 0.05), while mice in EP + BITC group showed increased time of visits and drinks compared with the EP group (*p* < 0.05). During reversed position learning, the number of visits and drinks decreased in EP group compared with the control group (*p* < 0.05), while the number of visits increased in EP + BITC group compared with the EP group (*p* < 0.05). In summary, the Intellicage learning tests indicate that the EP group experienced cognitive deficits, particularly in spatial learning and memory tasks. However, BITC treatment appears to mitigate these deficits, showing a potential cognitive-enhancing effect. These results provide valuable information for understanding the impact of the experimental conditions on cognitive function in the mice.

### 3.3 Morris water maze

**Figure 4:**
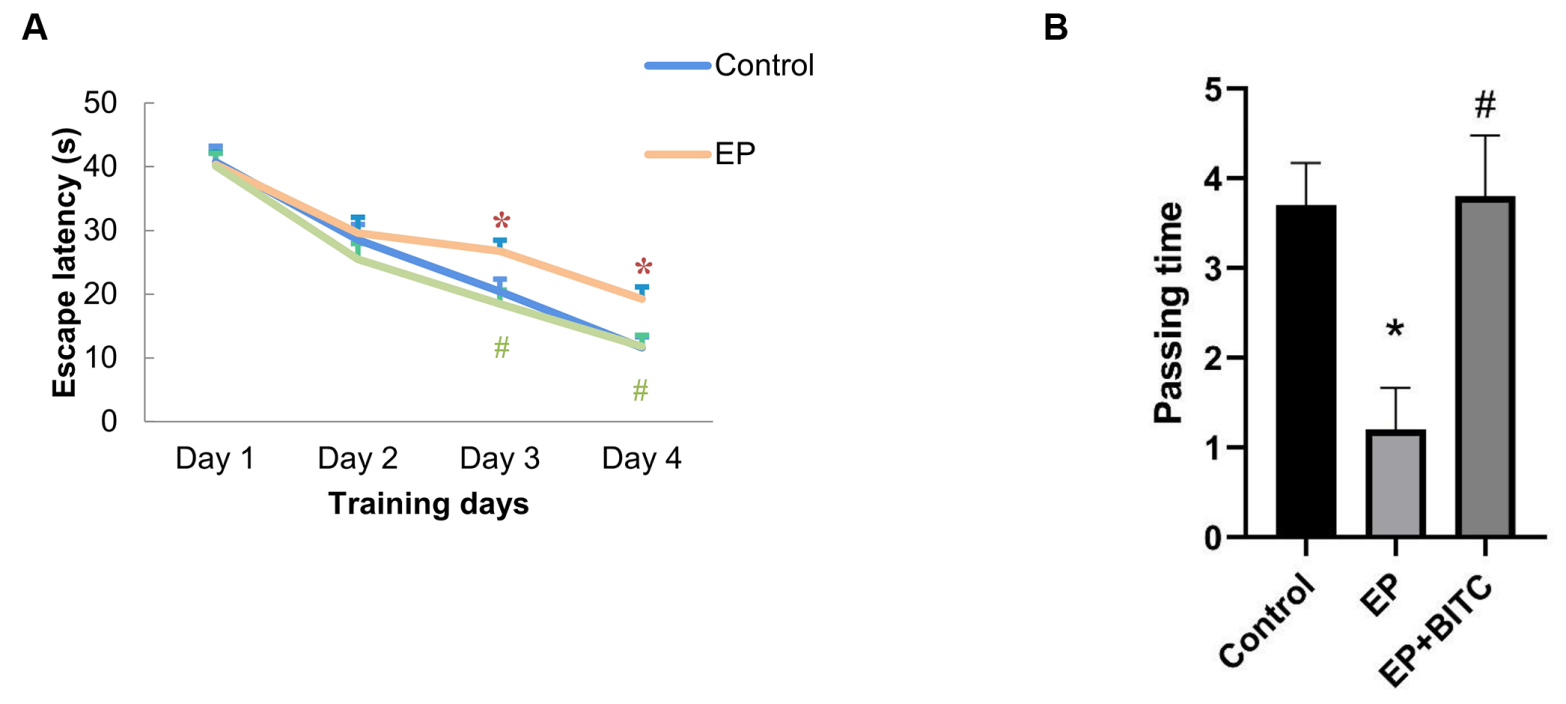
Morris water maze. Analysis of Morris water maze test in mice of control, EP, and EP + BITC (*n* = 10 per group; mean ± S.E.M.). **(A)** The escape latency of the three groups of mice during the 4 days of training. **(B)** The number of times the three groups of mice crossed the platform in the explore test. * Compared with control group: *p* < 0.05, # compared with EP group: *p* < 0.05. One-way ANOVA followed by *post hoc* least significant difference [LSD] multiple comparison tests.

In terms of latency in escape trials of Morris water maze (Figure 4), there was no difference in the escape latency between groups in the first 2 days (*p* > 0.05), form day 3 to day 4, mice in the EP group showed prolonged escape latency compared with mice in the control group (*p* < 0.05), while EP + BITC mice had shorter escape latency compared with the EP mice (*p* < 0.05). In probe trial, the passing time significantly reduced in the EP mice compared with the control mice (*p* < 0.05), while a remarkable increase of passing time was found in the EP + BITC mice compared with the EP mice without BITC (*p* < 0.05). In conclusion, the Morris water maze results indicate that the EP group experienced deficits in spatial learning and memory, particularly during later phases of training and in the probe trial. However, BITC treatment appears to ameliorate these deficits, suggesting a potential therapeutic effect on cognitive function in the context of spatial memory tasks.

### 3.4 Open field test

**Figure 5:**
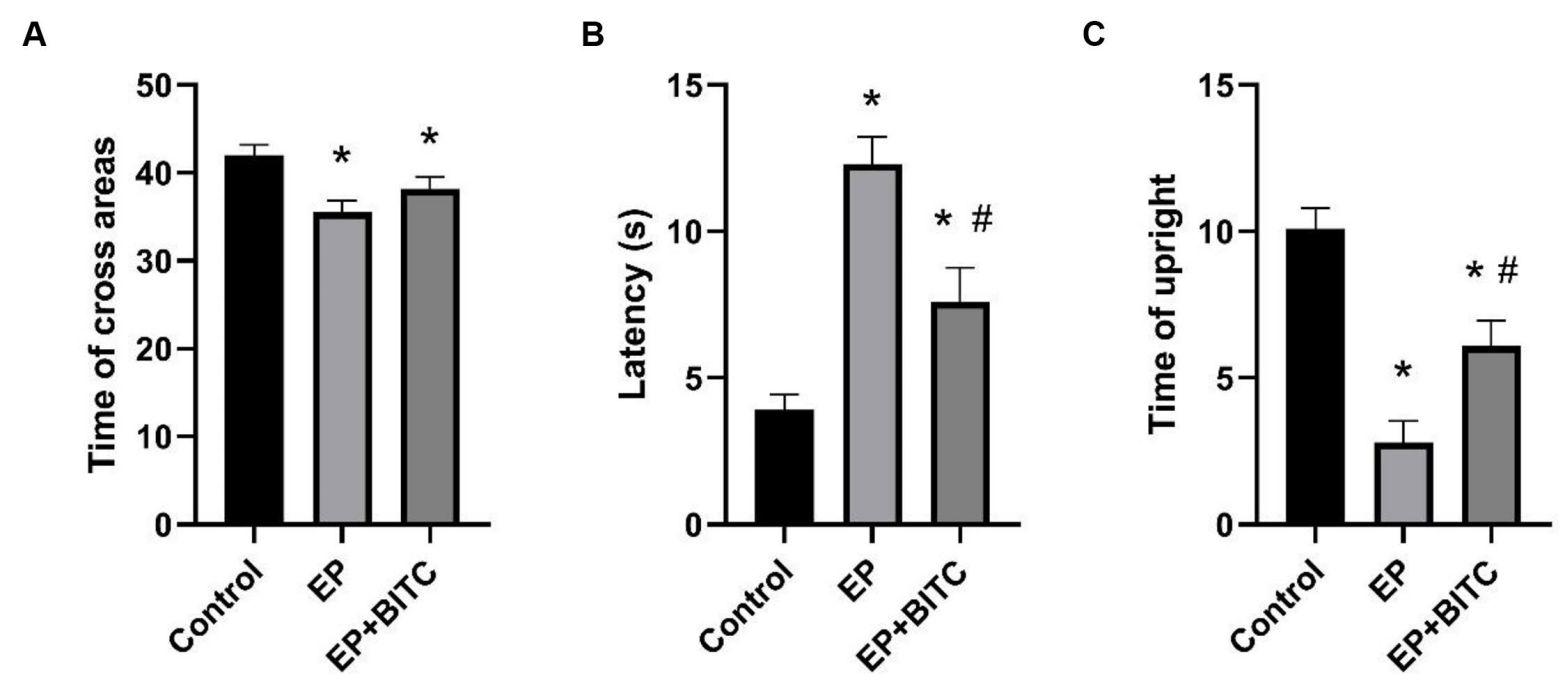
Open field test. Analysis of open field test in mice of control, EP, and EP + BITC (*n* = 10 per group; mean ± S.E.M.). **(A)** The three groups of mice crossed the areas times within 3 min. **(B)** The three groups of mice stayed in the central area within 3 min. **(C)** The upright times of three groups of mice within 3 min. * Compared with control group: *p* < 0.05, # compared with EP group: *p* < 0.05. One-way ANOVA followed by *post hoc* least significant difference [LSD] multiple comparison tests.

The results from open field test were shown in Figure 5. Compared with control, there was an obvious decrease in times of cross grids (*p* < 0.05), a longer latency in the central grid (*p* < 0.05), and a reduced times of upright (*p* < 0.05) in EP group. Meanwhile, a remarkable shorter latency in the central grid (*p* < 0.05) and an increased times of upright (*p* < 0.05) were observed in EP + BITC mice compared with EP mice. However, there is no difference in times of cross grids between EP with or without BITC (*p* > 0.05). To sum up, the open field test results suggest that the EP group exhibited altered exploratory and anxiety-like behaviors, while BITC treatment showed potential ameliorative effects. The anxiolytic-like effects of BITC are indicated by the improved performance in central grid latency and increased times of upright.

### 3.5 Step-down-type passive avoidance tests

**Figure 6:**
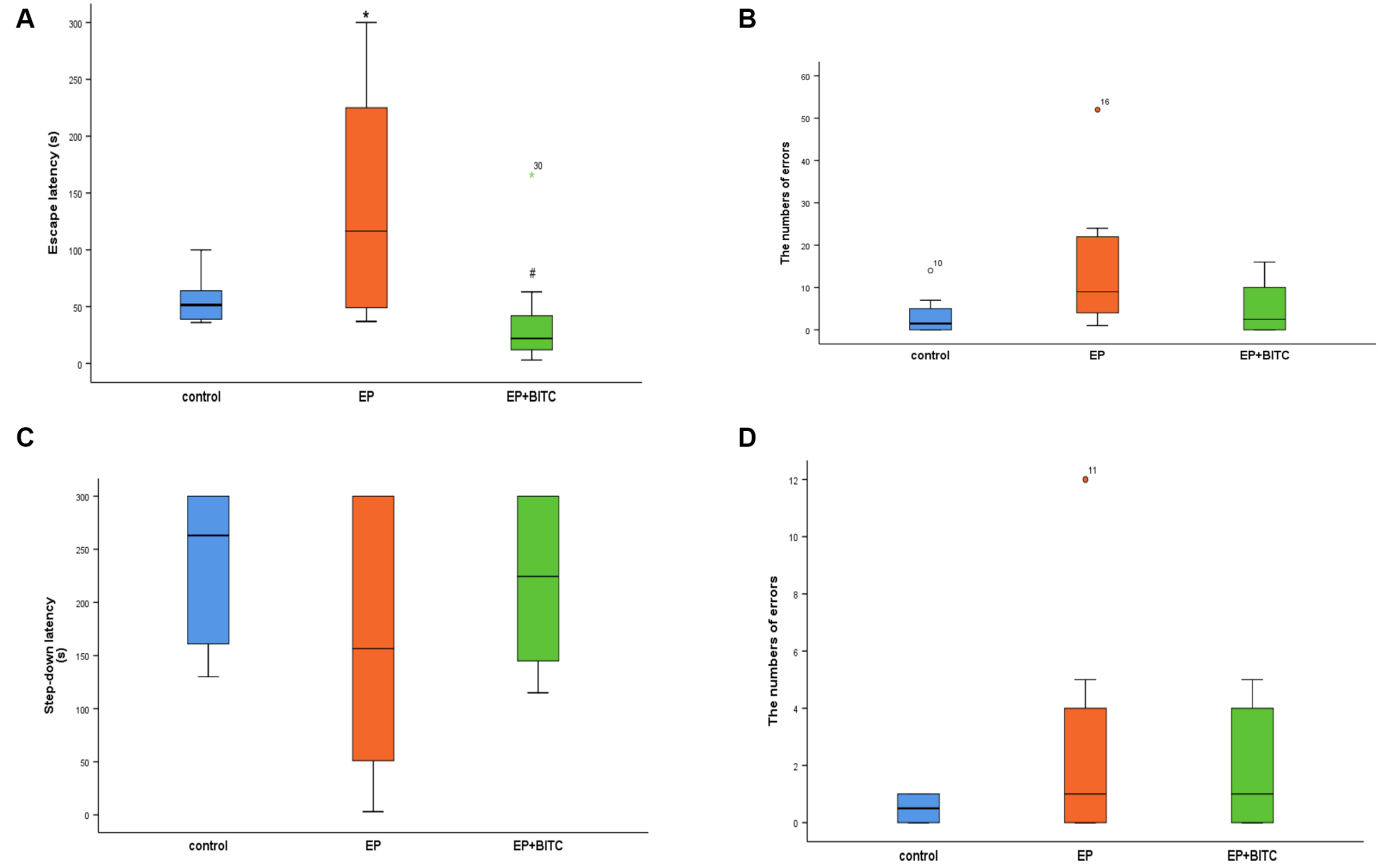
Step-down-type passive avoidance tests. Analysis of step-down-type passive avoidance tests in mice of control, EP, and EP + BITC (*n* = 10 per group; mean ± S.E.M.). **(A)** The escape latency of the three groups of mice during the training session. **(B)** The number of errors the three groups of mice jumped off the platform during the training session. **(C)** The Step-down latency of the three groups of mice during the retention session. **(D)** The number of errors the three groups of mice jumped off the platform during the retention session. * Compared with control group: *p* < 0.05, # compared with EP group: *p* < 0.05.; Analysis of significant differences using the Kruskar-Wallis test.

As shown in Figure 6, in training session, EP mice showed longer escape latency (*p* < 0.05), while the mice in EP + BITC group exhibited no difference compared with the controls (*p* > 0.05). However, there was no significant difference in the number of errors between these three groups (*p* > 0.05). In addition, in retention test, there is no remarkable difference between groups (*p* > 0.05). Considering the above, the Step-Down-Type Passive Avoidance Tests indicate that the EP mice initially struggled with learning, as indicated by longer escape latency, but this impairment was mitigated by BITC treatment. The comparable number of errors and performance in the retention test suggest that the quality of learning and memory retention was not significantly affected by the experimental conditions.

### 3.6 Brain–body ratio

**Figure 7:**
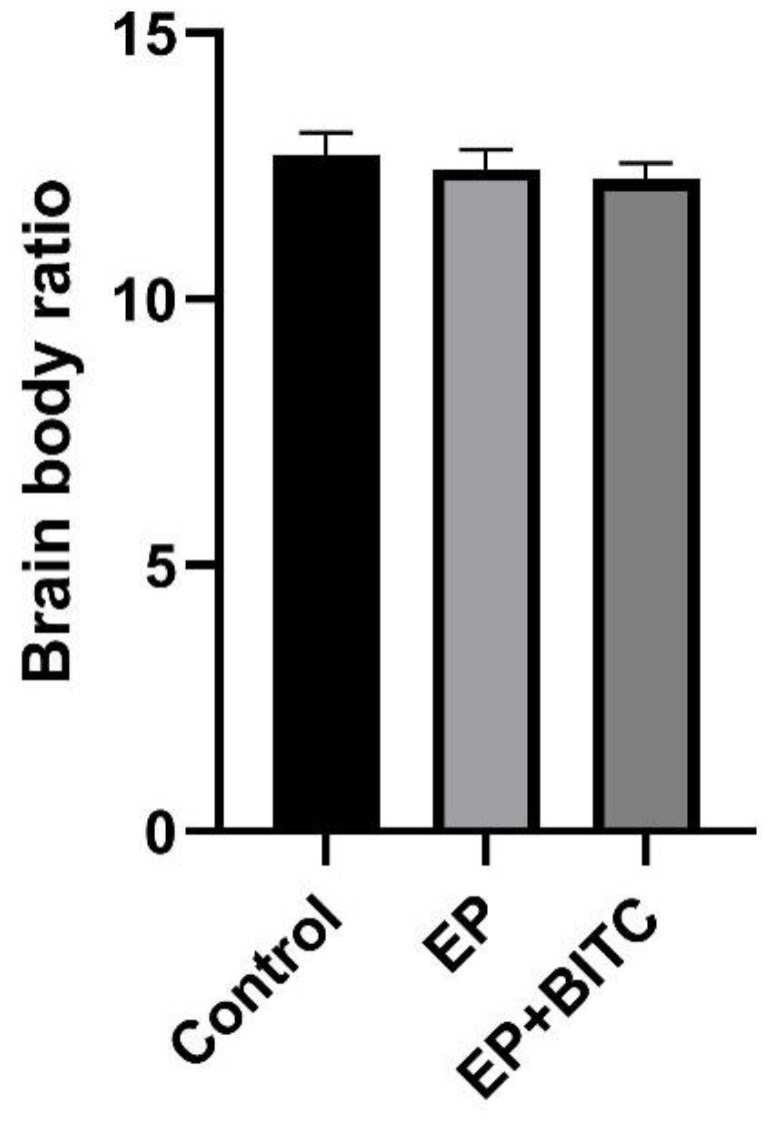
Brain-to-body weight ratio. Analysis of brain-to-body weight ratio in mice of control, EP, and EP + BITC. No difference between the control, EP, and EP + BITC groups (*n* = 10 per group; mean ± S.E.M.; *p* > 0.05. One-way ANOVA followed by *post hoc* least significant difference [LSD] multiple comparison tests).

During the treatment, no significant difference in body weights was observed between control, EP and EP + BITC mice (*p* > 0.05) (Figure 7). The brain-to-body ratio results showed the overall health stability of mice throughout the treatment, which was the basis for an accurate explanation of other experimental results and emphasized the safety of the interventions applied.

### 3.7 Morphological staining

**Figure 8:**
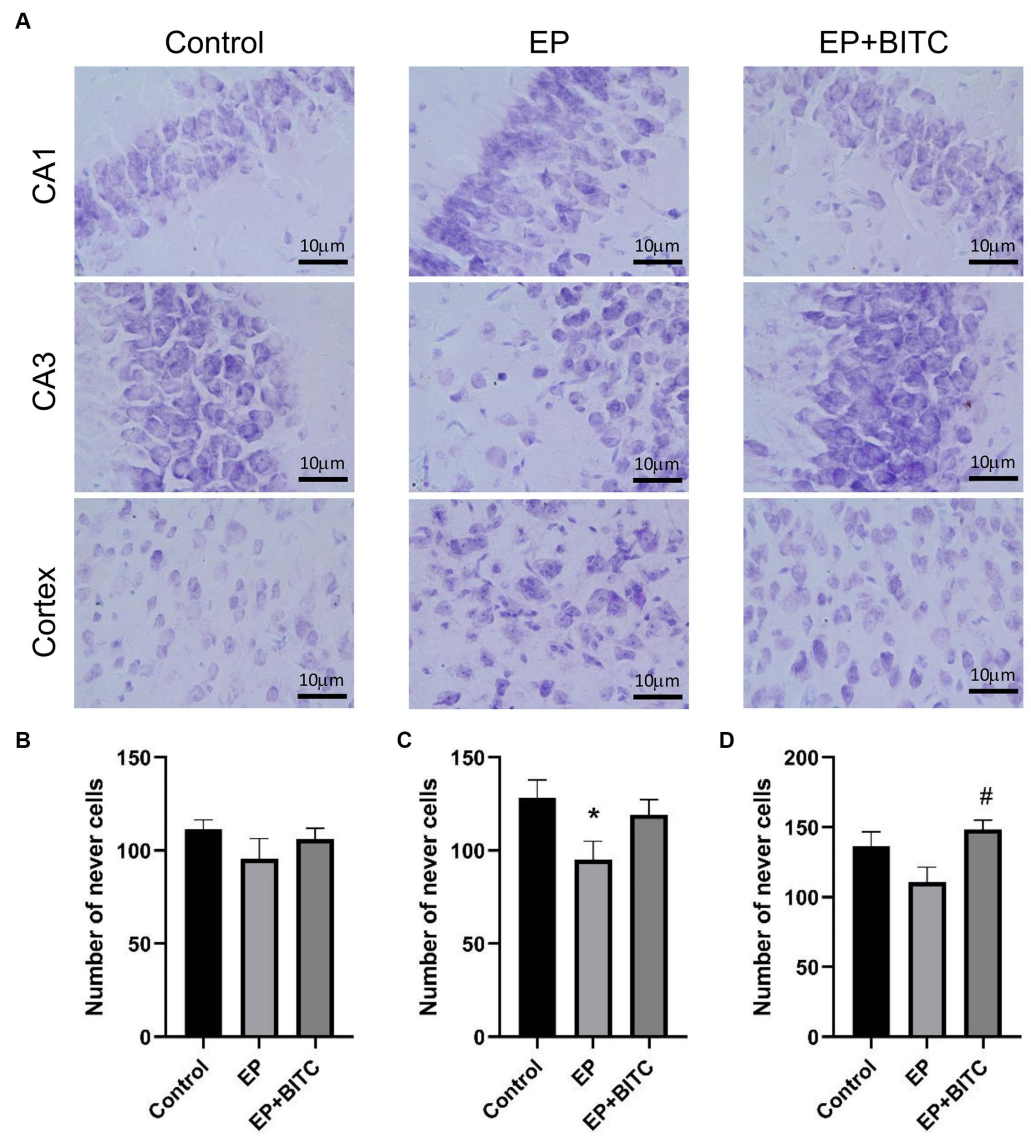
Nissl’s staining. Analysis of Nissl’s staining in mice of control, EP, and EP + BITC (*n* = 10 per group; mean ± S.E.M.). **(B)** The number of nerve cells in the CA1 region in the three groups of mice. No difference between the control, EP, and EP + BITC groups, *p* > 0.05. **(C)** The number of nerve cells in the CA3 region in the three groups of mice. **(D)** The number of nerve cells in the cortex region in the three groups of mice. * Compared with control group: *p* < 0.05, # compared with EP group: *p* < 0.05. One-way ANOVA followed by *post hoc* least significant difference [LSD] multiple comparison tests.

As shown in Immunofluorescence staining of NeuN (Supplementary Figures S2–S4), the mean fluorescence intensities of the hippocampal CA1 and CA3 regions in acute seizure model mice showed no significant difference (*p* > 0.05); compared with the control group and EP group, the mean fluorescence intensity of the cortex in the EP + BITC group decreased slightly (*p* < 0.05). As shown in Nissl’s staining of the chronic epilepsy state model (Figure 8), in terms of the number of cells in CA1 region of hippocampus, there was no difference between groups (*p* > 0.05). The EP mice showed decreased cells in CA3 compared with the control mice (*p* < 0.05). In addition, the number of cells in cortex was increased in EP + BITC group compared with the EP group (*p* < 0.05). These findings highlight the differential effects of epilepsy and BITC treatment on neuronal density in distinct brain regions. While epilepsy appears to induce neuronal loss in the CA3 region of the hippocampus, BITC treatment may exert neuroprotective effects, particularly evident in the cortex.

### 3.8 Real-time quantitative PCR

**Figure 9:**
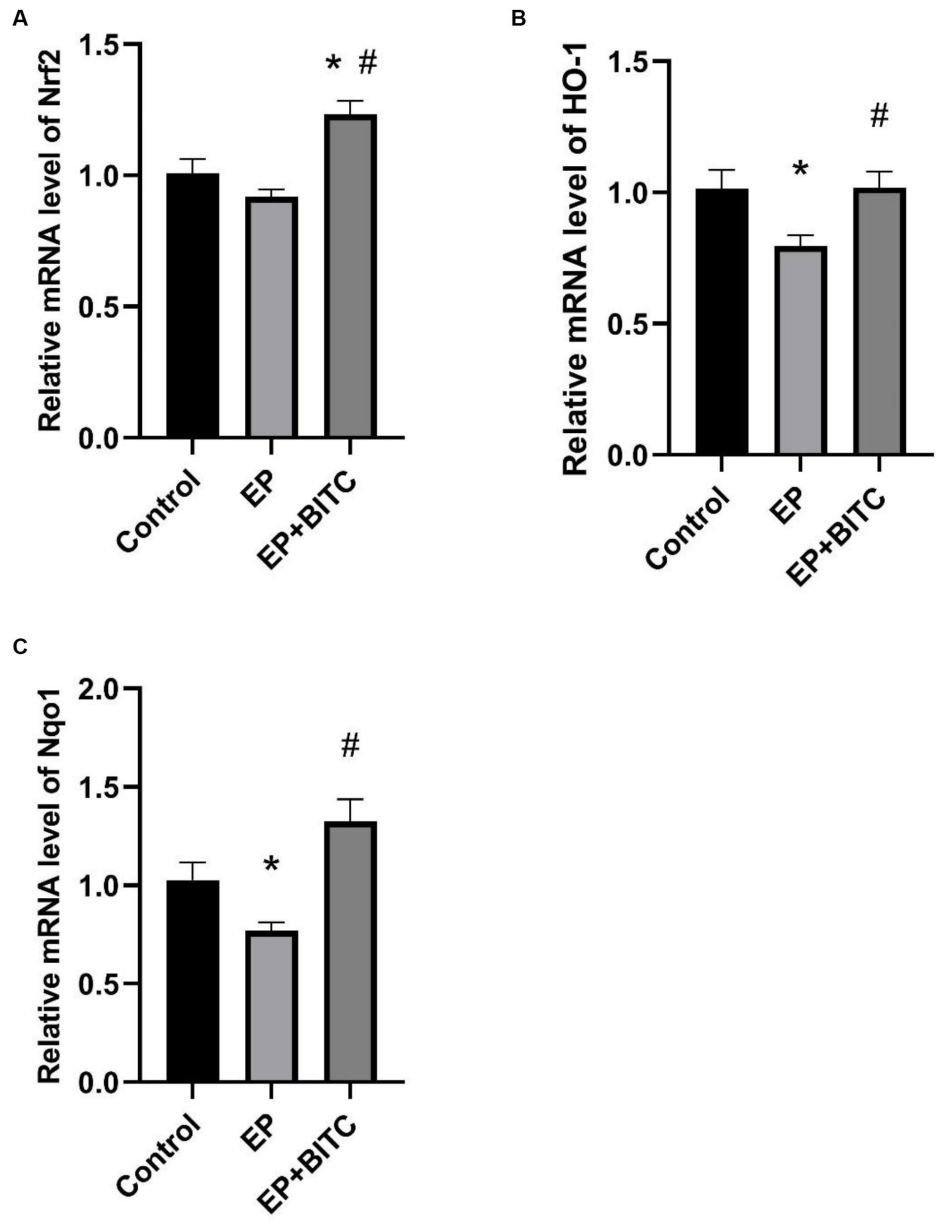
Analysis of mRNA levels of Nrf2, HO-1 and NQO1 from hippocampus between groups (*n* = 8 per group; mean ± S.E.M.). **(A)** Nrf2 transcription levels in hippocampus of three groups of mice. **(B)** HO-1 transcription levels in hippocampus of three groups of mice. **(C)** Nqo1 transcription levels in hippocampus of three groups of mice. * Compared with control group: *p* < 0.05, # compared with EP group: *p* < 0.05. One-way ANOVA followed by *post hoc* least significant difference [LSD] multiple comparison tests.

We investigated the transcription factor Nrf2, as well as two Nrf2-regulated enzymes, HO-1 and NQO1, in the transcriptional levels within the hippocampus. As shown in Figure 9, compared to the control group, the transcription levels of HO-1 and NQO1 in the EP group were significantly decreased (*p* < 0.05). Additionally, the treatment with BITC markedly increased the transcription levels of Nrf2, HO-1, and NQO1 in epileptic mice (*p* < 0.05). These results suggest that the antioxidant defense mechanism mediated by Nrf2 and its downstream targets may be damaged under epileptic conditions. The treatment of BITC enhanced the expression of Nrf2 and its downstream antioxidant enzymes, which potentially reduced oxidative stress and its harmful effects on hippocampal function in epileptic mice.

### 3.9 Determination of oxidative stress index in cerebral cortex of mice

**Figure 10:**
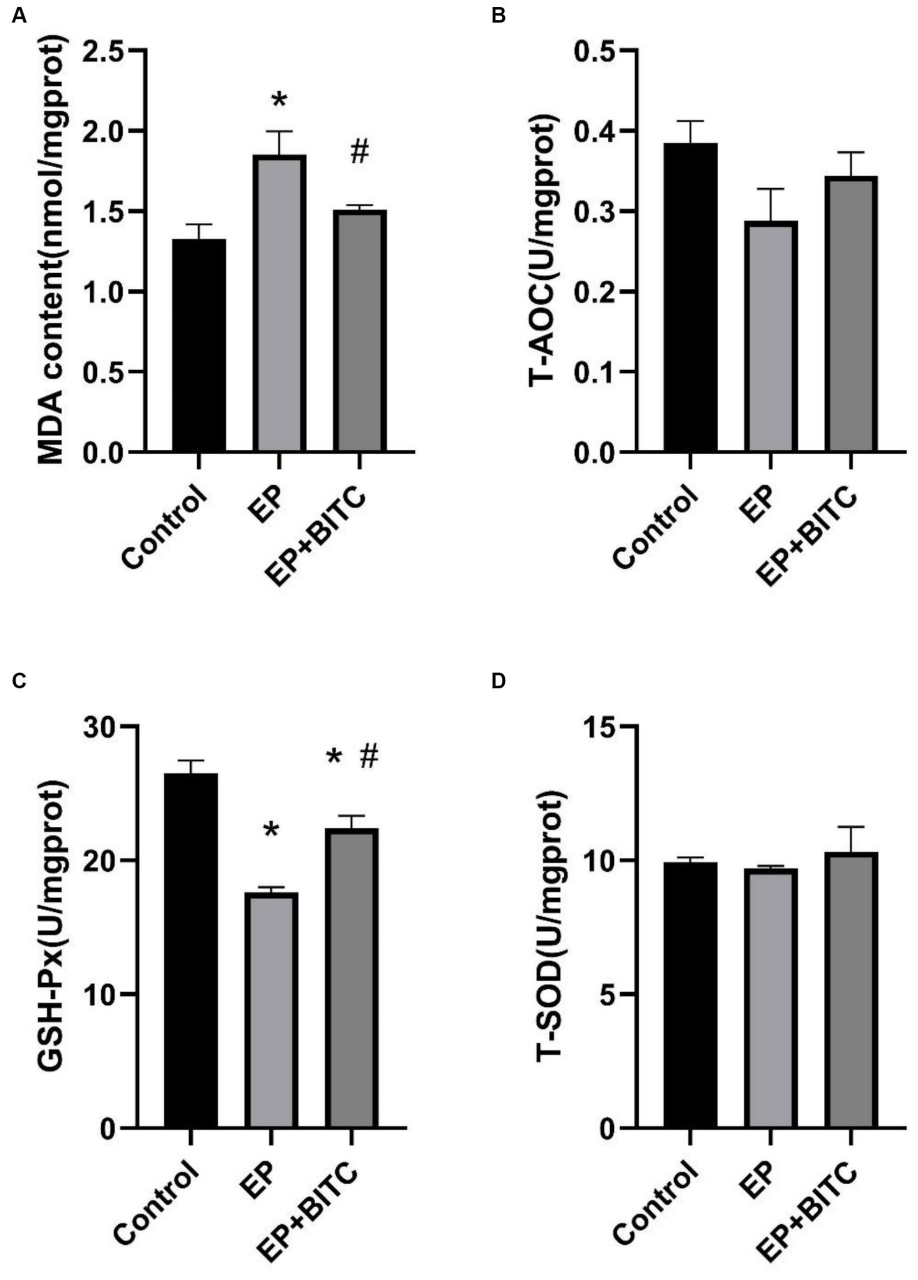
Analysis of MDA and anti-oxidative enzymes levels in cortex of mice between groups (*n* = 8 per group; mean ± S.E.M.). **(A)** The content of MDA in three groups of mice. **(B)** T-AOC activity of three groups of mice. No difference between the control, EP, and EP + BITC groups, *p* > 0.05. **(C)** GSH-Px activity of three groups of mice. **(D)** T-SOD activity of three groups of mice. No difference between the control, EP, and EP + BITC groups, *p* > 0.05. * Compared with control group: *p* < 0.05, # compared with EP group: *p* < 0.05. One-way ANOVA followed by *post hoc* least significant difference [LSD] multiple comparison tests.

In our study, we assessed the MDA levels and the activity of antioxidant enzymes in the cerebral cortex across various mouse groups. Figure 10 illustrates that, relative to the control group, the MDA levels in the EP group were elevated, while GSH-Px activity diminished (*p* < 0.05). Conversely, in the BITC intervention group, MDA levels decreased, and GSH-Px activity increased when compared with the EP group (*p* < 0.05). Furthermore, the activities of T-AOC and SOD did not show significant differences across the groups. BITC might enhance the antioxidant defense mechanism, potentially by upregulating GSH-Px activity, thereby reducing lipid peroxidation as indicated by lower MDA levels. This implies that BITC could have therapeutic potential in mitigating oxidative stress-related neuronal damage in epilepsy.

## 4 Discussion

TLE is the most common form of intractable epilepsy (22). In clinical studies, TLE patients have been shown to have cognitive impairments (23). Chronic central nervous system dysfunction is usually accompanied with persistent and recurrent seizures (24). Reactive oxygen species (ROS) formation and consequent oxidative stress (OS) are reported to show a potential role in seizures and closely linked to neuroinflammation (25, 26). BITC, an extract of cruciferous vegetables, has been shown to have anti-inflammatory and antioxidative stress effects, as well as cytoprotective effects (16, 27). In the present study, we demonstrate that BITC exhibits improvements in cognitive dysfunction and neuroprotective effects in lithium pilocarpine-induced epileptic mice.

The lithium-pilocarpine-induced epilepsy model represents a durable, reliable, and cost-effective method with a short timeframe. Pilocarpine activates the M1 receptor, elevates glutamate levels, triggers NMDA receptor activation in the hippocampus, and induces hippocampal neuronal damage (28, 29). This model reproduces the majority of the clinical neuropathological features of TLE and closely resembles persistent epilepsy in humans (30). Sandro et al. found that long-term memory impairment in rats having early lithium-pilocarpine-induced epilepsy during object recognition tasks (31). Jia et al. used male C57BL/6 J mice induced status epilepticus (SE) through pilocarpine hydrochloride injection (320 mg/kg) and conducted behavioral testing within 7–10 days post-surgery. SE reduced the recognition index of new and old objects in mice, leading to increased time spent in the central area during open field tests (32). Wu et al. performed the Morris water maze test on a lithium-pilocarpine-induced epileptic rat model, revealing a significant impairment in spatial memory function (33). The modeling method used in our experiment is similar to that of other experiments, and the results of the behavioral tests are consistent. Four different behavioral detection methods were used in this study. Intellicage learning tests can protect experimental animals from human interference, truly reflect the learning and memory abilities of experimental animals in their natural state and monitor the behavior of multiple experimental animals at the same time, unaffected by time series and other factors (34). Morris water maze is a classical behavioral experiment to study and evaluate the spatial learning and memory abilities of animals (9). Open field test can measure the anxiety, spontaneous activity, and exploratory behavior of experimental animals; Step-down-type passive avoidance tests are based on the fact that mice tend to jump on small platforms that are uncomfortable, and animals inhibit their behavior to avoid electric shocks, which is measured by longer delays or refusing to step down. Delay is used to evaluate memory. In our investigation, compared with the control group, epileptic mice exhibited a reduction in corner visits and drinking episodes, an extended escape latency, diminished occurrences of crossing the original platform, grid crossing, and standing frequency. Meanwhile, treatment with BITC had a positive effect on spatial cognition, exploratory behavior, and learning and memory abilities in epileptic mice, with a significant enhancement in spatial cognition and learning and memory abilities.

The hippocampus plays an important role in spatial working memory (35). Chen et al. used Timm staining and transmission electron microscopy to observe the effect of pilocarpine-induced epilepsy on the CA3 region of the hippocampus in rats, and they observed abnormal mossy fiber sprouting (MFS) and vacuolar degeneration in the CA3 region of the model group (36). Alberto et al. used N-acetylcysteine and sulforaphane (SFN) as a temporary treatment for the epileptic rats and the results showed that it reduced the loss of hippocampal neurons and ameliorated cognitive impairments (37). Nissl staining can indicate the Nissl body of pyramidal cells in purple, and by observing the number of Nissl bodies, we can judge the damage in brain nerve cells and then the cognitive function of the brain. In our study, consistent with previous research, BITC treatment improved the number of neurons in the cortex and CA3 region of the hippocampus in epileptic mice and prevented the loss of neurons.

Cruciferous vegetables are recognized as abundant sources of isothiocyanates (ITCs), including sulforaphane (SFN) and BITC (38). These compounds demonstrate the capability to activate the Nrf2 pathway, associated with antioxidant and anti-inflammatory effects (39). The Nrf2 pathway plays a key role in up-regulating gene expression driven by antioxidant response elements (ARE), thereby reducing inflammation and providing cellular protection against oxidative stress (40, 41). Furthermore, the protective effects of Nrf2 and its signaling pathway have been confirmed in many disease models, such as vinblastine improving methotrexate-induced nephrotoxicity through Nrf2/HO-1 antioxidant pathway (42). These findings suggest that a targeted approach to Nrf2 holds therapeutic promise in conditions linked to oxidative stress and inflammation. In our experiment, BITC treatment increased the mRNA transcription levels of HO-1, NQO1, and Nrf2 in epileptic mice. This suggests that BITC may target the activation of Nrf2/HO-1 signal pathway in epileptic mice, play antioxidant and anti-inflammatory effects, and then protect hippocampal neurons from lithium pilocarpine-induced damage and improve cognitive impairment.

In summary, BITC exhibits neuroprotective effects on pilocarpine-induced epileptic mice. Treatment with BITC enhances the antioxidant capacity of the brain tissue in epileptic mice, improving cognitive impairments and neuronal damage caused by seizures. Our research provides scientific evidence that BITC is a candidate drug for the prevention and treatment of epilepsy and has great potential and value.

## 5 Limitations of our study

Due to limitations in funding and data recording, our research showed that BITC had no significant impact during the acute phase of epileptic seizures. However, we believe that BITC’s potential in alleviating oxidative stress may provide neuroprotective effects in the chronic stages of epilepsy, emphasizing the need for further research to explore its full therapeutic potential. We highlight the critical role of oxidative stress in neurological diseases, including epilepsy, and the promising antioxidant capabilities of BITC. Although cognitive impairment often co-occurs with epilepsy, the complex interplay between oxidative stress, cognitive dysfunction, and epilepsy indicates potential therapeutic strategies worthy of in-depth investigation. Our goal is to extend our research through comparative analysis of BITC’s effects in different epilepsy models, such as the PTZ model, to comprehensively understand its neuroprotective efficacy. Additionally, we plan to conduct seizure threshold tests in both pilocarpine and PTZ models to quantitatively assess BITC’s neuroprotective abilities and investigate its mechanism of action, with a focus on its modulation of oxidative stress.

## Data availability statement

The raw data supporting the conclusions of this article will be made available by the authors, without undue reservation.

## Ethics statement

The animal study was approved by Ningxia Medical University Experimental Animal Center (IACUC Animal code: SCXK (Ning) 2017-0001) The Animal Care and Use Committee of Ningxia Medical University approved the experiment (No. NXMU2020-021). The study was conducted in accordance with the local legislation and institutional requirements.

## Author contributions

CX: Writing – review & editing. ZH: Writing – original draft. PN: Writing – original draft. GT: Writing – original draft. GoY: Writing – original draft. GuY: Methodology, Writing – original draft. LH: Methodology, Writing – original draft. ML: Methodology, Writing – original draft. WH: Methodology, Writing – original draft. WY: Supervision, Writing – review & editing. ZR: Writing – review & editing.

## References

[1] Fisher RS Bonner AM. The revised definition and classification of epilepsy for neurodiagnostic technologists. Neurodiagnostic J. (2018) 58:1–10. doi: 10.1080/21646821.2018.1428455 29562876

[2] Janmohamed M Brodie MJ Kwan P. Pharmacoresistance – epidemiology, mechanisms, and impact on epilepsy treatment. Neuropharmacology. (2020) 168:107790. doi: 10.1016/j.neuropharm.2019.107790 31560910

[3] Milovanovic JR Jankovic SM Milovanovic D Ruzic ZD Folic M Kostic M. Contemporary surgical management of drug-resistant focal epilepsy. Expert Rev Neurother. (2020) 20:23–40. doi: 10.1080/14737175.2020.1676733 31583915

[4] Carron S Dezsi G Ozturk E Nithianantharajah J Jones NC. Cognitive deficits in a rat model of temporal lobe epilepsy using touchscreen-based translational tools. Epilepsia. (2019) 60:1650–60. doi: 10.1111/epi.16291 31335966

[5] Dey S Dubey V Dixit AB Tripathi M Chandra PS Banerjee J. Differential levels of tryptophan–kynurenine pathway metabolites in the hippocampus, anterior temporal lobe, and neocortex in an animal model of temporal lobe epilepsy. Cells. (2022) 11:3560. doi: 10.3390/cells11223560 36428989 PMC9688794

[6] Johnson EL Adams JN Solbakk AK Endestad T Larsson PG Ivanovic J. Dynamic frontotemporal systems process space and time in working memory. PLoS Biol. (2018) 16:e2004274. doi: 10.1371/journal.pbio.2004274 29601574 PMC5895055

[7] Chang YA Marshall A Bahrami N Mathur K Javadi SS Reyes A. Differential sensitivity of structural, diffusion, and resting-state functional mri for detecting brain alterations and verbal memory impairment in temporal lobe epilepsy. Epilepsia. (2019) 60:935–47. doi: 10.1111/epi.14736 31020649 PMC6584028

[8] Wang M Zhang X Jia W Zhang C Boczek T Harding M. Circulating glutathione peroxidase and superoxide dismutase levels in patients with epilepsy: a meta-analysis. Seizure. (2021) 91:278–86. doi: 10.1016/j.seizure.2021.07.001 34252880

[9] Higaki A Mogi M Iwanami J Min LJ Bai HY Shan BS. Predicting outcome of Morris water maze test in vascular dementia mouse model with deep learning. PLoS One. (2018) 13:e191708. doi: 10.1371/journal.pone.0191708 PMC5802845 29415035

[10] Tsybina Y Kastalskiy I Krivonosov M Zaikin A Kazantsev V Gorban AN. Astrocytes mediate analogous memory in a multi-layer neuron–astrocyte network. Neural Comput & Applic. (2022) 34:9147–60. doi: 10.1007/s00521-022-06936-9

[11] Kessels R Bergmann HC. What does the hippocampus do during working-memory tasks? A cognitive-neuropsychological perspective. Cogn Neurosci. (2022) 13:210–1. doi: 10.1080/17588928.2022.2131745 36218275

[12] Esteve M. Mechanisms underlying biological effects of cruciferous Glucosinolate-derived isothiocyanates/indoles: a focus on metabolic syndrome. Front Nutr. (2020) 7:111–1. doi: 10.3389/fnut.2020.00111 32984393 PMC7492599

[13] El BS Ogaly HA Abd-Elsalam RM Azouz AA. Benzyl isothiocyanates modulate inflammation, oxidative stress, and apoptosis via nrf2/ho-1 and nf-kappab signaling pathways on indomethacin-induced gastric injury in rats. Food Funct. (2021) 12:6001–13. doi: 10.1039/d1fo00645b 34037056

[14] Li Z Wu H Liu J Hao H Bi J Hou H. Synergistic effects of benzyl isothiocyanate and resveratrol against listeria monocytogenes and their application in chicken meat preservation. Food Chem. (2023) 419:135984. doi: 10.1016/j.foodchem.2023.135984 37044056

[15] Mitra S Emran TB Chandran D Zidan BMRM Das R Mamada SS. Cruciferous vegetables as a treasure of functional foods bioactive compounds: targeting p53 family in gastrointestinal tract and associated cancers. Front Nutr. (2022) 9:951935. doi: 10.3389/fnut.2022.951935 35990357 PMC9386315

[16] Chuang WT Yen CC Huang CS Chen HW Lii CK. Benzyl isothiocyanate ameliorates high-fat diet-induced hyperglycemia by enhancing nrf2-dependent antioxidant defense-mediated irs-1/akt/tbc1d1 signaling and glut4 expression in skeletal muscle. J Agric Food Chem. (2020) 68:15228–38. doi: 10.1021/acs.jafc.0c06269 33301311

[17] Ibrahim A Al-Hizab FA Abushouk AI Abdel-Daim MM. Nephroprotective effects of benzyl isothiocyanate and resveratrol against cisplatin-induced oxidative stress and inflammation. Front Pharmacol. (2018) 9:1268. doi: 10.3389/fphar.2018.01268 30524274 PMC6258716

[18] Peluso I Palmery M. Editorial: therapeutic index for nutraceuticals in inflammation-related diseases: efficacy, bioavailability, metabolism and interactions with drugs. Front Pharmacol. (2020) 11:61. doi: 10.3389/fphar.2020.00061 32116728 PMC7031407

[19] Wu N Wang F Jin Z Zhang Z Wang LK Zhang C. Effects of gaba(b) receptors in the insula on recognition memory observed with intellicage. Behav Brain Funct. (2017) 13:7. doi: 10.1186/s12993-017-0125-4 28416021 PMC5392977

[20] Zhang R Miao QW Zhu CX Zhao Y Liu L Yang J. Sulforaphane ameliorates neurobehavioral deficits and protects the brain from amyloid beta deposits and peroxidation in mice with Alzheimer-like lesions. Am J Alzheimers Dis Other Dement. (2015) 30:183–91. doi: 10.1177/1533317514542645 PMC10852928 25024455

[21] Zhang R Zhang J Fang L Li X Zhao Y Shi W. Neuroprotective effects of sulforaphane on cholinergic neurons in mice with Alzheimer’s disease-like lesions. Int J Mol Sci. (2014) 15:14396–410. doi: 10.3390/ijms150814396 25196440 PMC4159858

[22] Yakimov AM Timechko EE Areshkina IG Usoltseva AA Yakovleva KD Kantimirova EA. MicroRNAs as biomarkers of surgical outcome in mesial temporal lobe epilepsy: a systematic review. Int J Mol Sci. (2023) 24:5694. doi: 10.3390/ijms24065694 36982768 PMC10052204

[23] Giovagnoli AR Franceschetti S Reati F Parente A Maccagnano C Villani F. Theory of mind in frontal and temporal lobe epilepsy: cognitive and neural aspects. Epilepsia. (2011) 52:1995–2002. doi: 10.1111/j.1528-1167.2011.03215.x 21883176

[24] Lopez-Rivera JA Smuk V Leu C Nasr G Vegh D Stefanski A. Incidence and prevalence of major epilepsy-associated brain lesions. Epilepsy Behav Rep. (2022) 18:100527. doi: 10.1016/j.ebr.2022.100527 35243289 PMC8885987

[25] Geronzi U Lotti F Grosso S. Oxidative stress in epilepsy. Expert Rev Neurother. (2018) 18:427–34. doi: 10.1080/14737175.2018.1465410 29651881

[26] Paudel YN Shaikh MF Shah S Kumari Y Othman I. Role of inflammation in epilepsy and neurobehavioral comorbidities: implication for therapy. Eur J Pharmacol. (2018) 837:145–55. doi: 10.1016/j.ejphar.2018.08.020 30125565

[27] Park WS Lee J Na G Park S Seo S Choi JS. Benzyl isothiocyanate attenuates inflammasome activation in *Pseudomonas aeruginosa* LPS-stimulated THP-1 cells and exerts regulation through the MAPKs/NF-κB pathway. Int J Mol Sci. (2022) 23:1228. doi: 10.3390/ijms23031228 35163151 PMC8835927

[28] Kraus KL Nawreen N Godale CM Chordia AP Packard B LaSarge CL. Hippocampal glucocorticoid receptors modulate status epilepticus severity. Neurobiol Dis. (2023) 178:106014. doi: 10.1016/j.nbd.2023.106014 36702319 PMC10055427

[29] Meller S Brandt C Theilmann W Klein J Loscher W. Commonalities and differences in extracellular levels of hippocampal acetylcholine and amino acid neurotransmitters during status epilepticus and subsequent epileptogenesis in two rat models of temporal lobe epilepsy. Brain Res. (2019) 1712:109–23. doi: 10.1016/j.brainres.2019.01.034 30703372

[30] André V Dubé C François J Leroy C Rigoulot MA Roch C. Pathogenesis and pharmacology of epilepsy in the lithium-pilocarpine model. Epilepsia. (2007) 48:41–7. doi: 10.1111/j.1528-1167.2007.01288.x 17910580

[31] Cordova SD Loss CM de Oliveira DL. Low-intensity physical training recovers object recognition memory impairment in rats after early-life induced status epilepticus. Int J Dev Neurosci. (2013) 31:196–201. doi: 10.1016/j.ijdevneu.2013.01.002 23318691

[32] Jia X Wang Q Ji J Lu W Liu Z Tian H. Mitochondrial transplantation ameliorates hippocampal damage following status epilepticus. Anim Model Exp Med. (2023) 6:41–50. doi: 10.1002/ame2.12310 PMC9986225 36734302

[33] Wu J Wang L Huang Y Wu Q Luo X Li Y. Cognitive impairment and mossy fiber sprouting in a rat model of drug-resistant epilepsy induced by lithium-pilocarpine. Curr Neurovasc Res. (2021) 18:374–80. doi: 10.2174/1567202618666210917155408 34538230

[34] Kiryk A Janusz A Zglinicki B Turkes E Knapska E Konopka W. Intellicage as a tool for measuring mouse behavior - 20 years perspective. Behav Brain Res. (2020) 388:112620. doi: 10.1016/j.bbr.2020.112620 32302617

[35] Song D Wang D Yang Q Yan T Wang Z Yan Y. The lateralization of left hippocampal ca3 during the retrieval of spatial working memory. Nat Commun. (2020) 11:2901. doi: 10.1038/s41467-020-16698-4 32518226 PMC7283476

[36] Jia C Zhang R Wei L Xie J Zhou S Yin W. Investigation of the mechanism of tanshinone iia to improve cognitive function via synaptic plasticity in epileptic rats. Pharm Biol. (2023) 61:100–10. doi: 10.1080/13880209.2022.2157843 36548216 PMC9788714

[37] Pauletti A Terrone G Shekh-Ahmad T Salamone A Ravizza T Rizzi M. Targeting oxidative stress improves disease outcomes in a rat model of acquired epilepsy. Brain. (2019) 142:e39. doi: 10.1093/brain/awz130 31145451 PMC6598637

[38] Kim SY Park JE Kim EO Lim SJ Nam EJ Yun JH. Exposure of kale root to nacl and na(2) seo(3) increases isothiocyanate levels and nrf2 signalling without reducing plant root growth. Sci Rep. (2018) 8:3999. doi: 10.1038/s41598-018-22411-9 29507323 PMC5838157

[39] Kubo E Chhunchha B Singh P Sasaki H Singh DP. Sulforaphane reactivates cellular antioxidant defense by inducing nrf2/are/prdx6 activity during aging and oxidative stress. Sci Rep. (2017) 7:14130. doi: 10.1038/s41598-017-14520-8 29074861 PMC5658327

[40] Albarakati A Baty RS Aljoudi AM Habotta OA Elmahallawy EK Kassab RB. Luteolin protects against lead acetate-induced nephrotoxicity through antioxidant, anti-inflammatory, anti-apoptotic, and nrf2/ho-1 signaling pathways. Mol Biol Rep. (2020) 47:2591–603. doi: 10.1007/s11033-020-05346-1 32144527

[41] McMahon M Itoh K Yamamoto M Hayes JD. Keap1-dependent proteasomal degradation of transcription factor nrf2 contributes to the negative regulation of antioxidant response element-driven gene expression. J Biol Chem. (2003) 278:21592–600. doi: 10.1074/jbc.M300931200 12682069

[42] Shalaby YM Menze ET Azab SS Awad AS. Involvement of nrf2/ho-1 antioxidant signaling and nf-κb inflammatory response in the potential protective effects of vincamine against methotrexate-induced nephrotoxicity in rats: cross talk between nephrotoxicity and neurotoxicity. Arch Toxicol. (2019) 93:1417–31. doi: 10.1007/s00204-019-02429-2 31020375

