# Supplementary material for: Benzyl isothiocyanate ameliorates cognitive function in mice of chronic temporal lobe epilepsy
Source: Front Neurol. 2024 Apr 23;15:1330102. doi: 10.3389/fneur.2024.1330102 (PMC11074416; doi:10.3389/fneur.2024.1330102)
Supplement: Supplementary file 1 [file Data_Sheet_1.pdf]

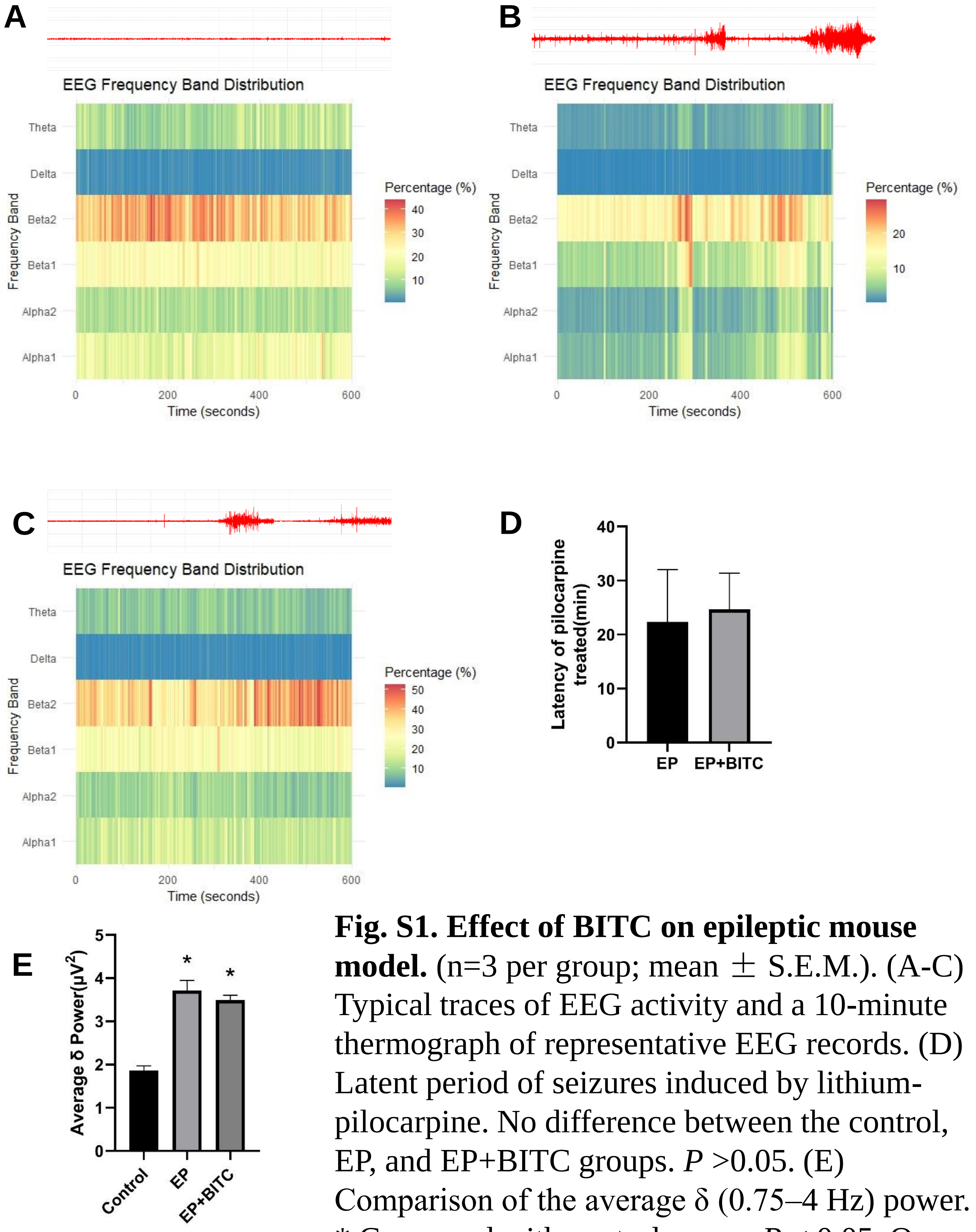

Fig. S1. Effect of BITC on epileptic mouse model. (n=3 per group; mean ± S.E.M.). (A-C) Typical traces of EEG activity and a 10-minute thermograph of representative EEG records. (D) Latent period of seizures induced by lithium-pilocarpine. No difference between the control, EP, and EP+BITC groups. P >0.05. (E) Comparison of the average δ (0.75–4 Hz) power. * Compared with control group: P < 0.05. One-way ANOVA followed by post hoc least significant difference [LSD] multiple comparison tests.

A

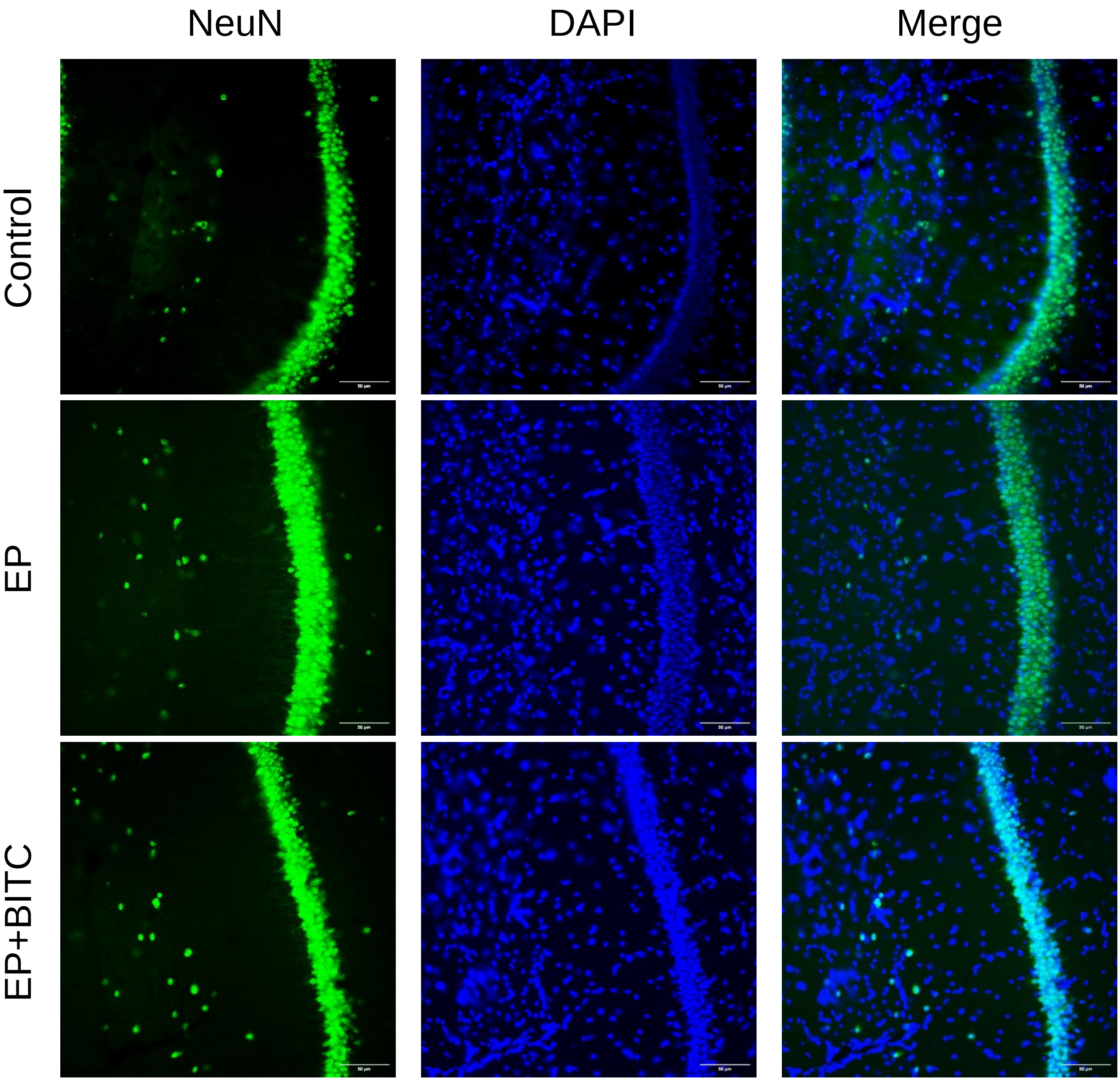

B

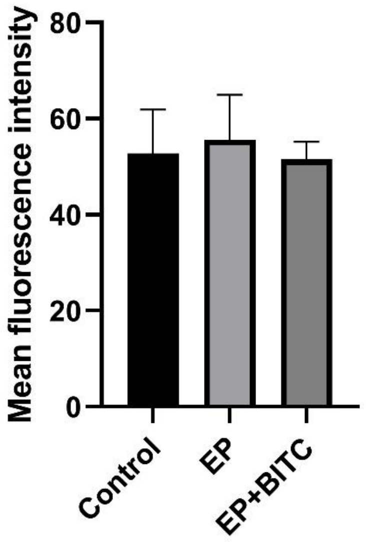

Fig. S2. Immunofluorescence staining of NeuN in the hippocampal CA1 region. (n=3 per group; mean ± S.E.M.). (B) Mean fluorescence intensity in the CA1 region in the three groups of mice. No difference between the control, EP, and EP+BITC groups. *P* >0.05. One-way ANOVA followed by post hoc least significant difference [LSD] multiple comparison tests.

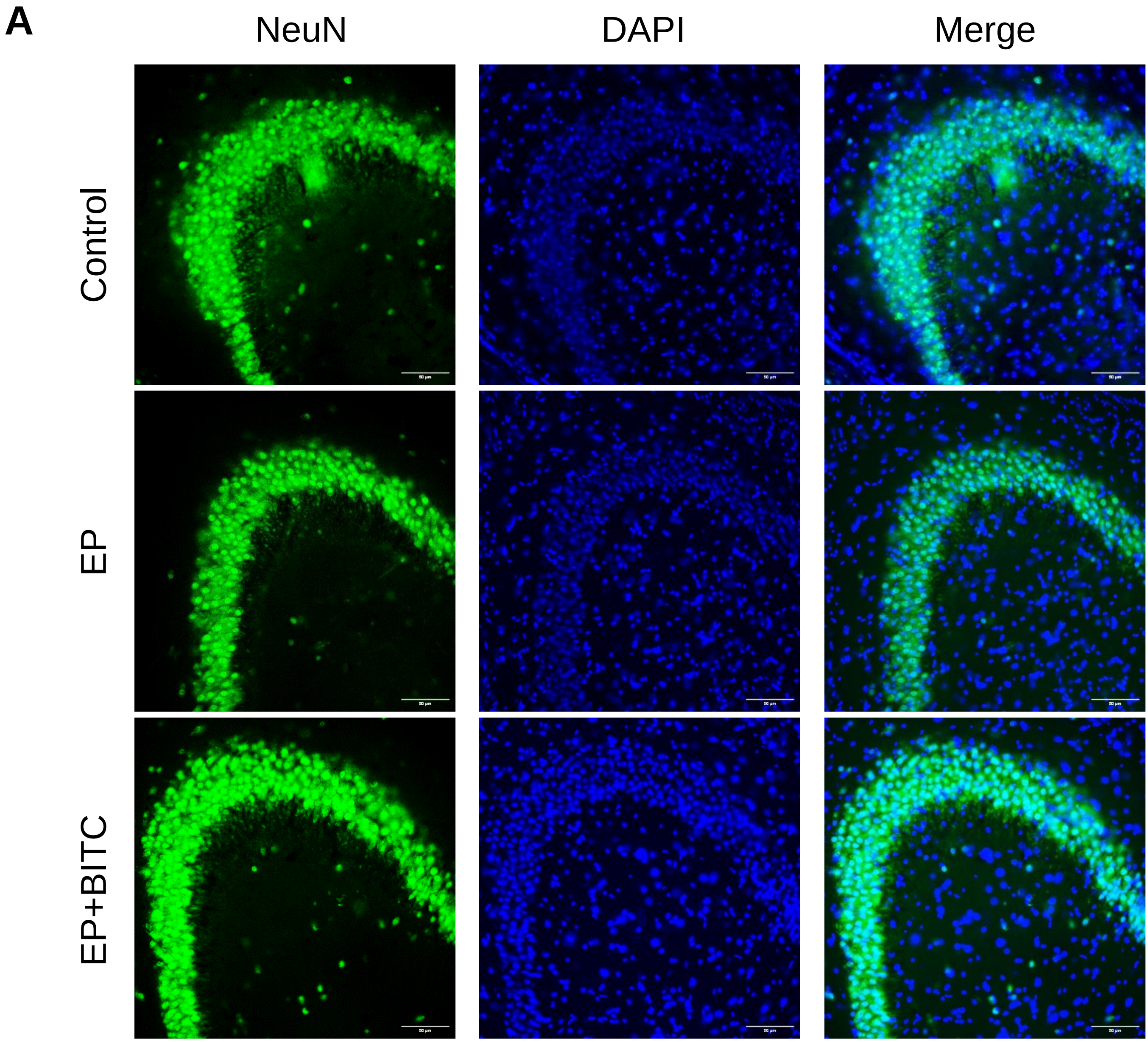

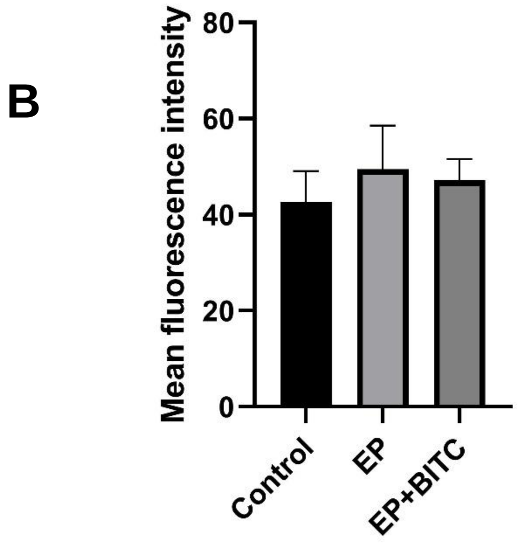

Fig. S3. Immunofluorescence staining of NeuN in the hippocampal CA3 region. (n=3 per group; mean ± S.E.M.). (B) Mean fluorescence intensity in the CA3 region in the three groups of mice. No difference between the control, EP, and EP+BITC groups. *P* >0.05. One-way ANOVA followed by post hoc least significant difference [LSD] multiple comparison tests.

A

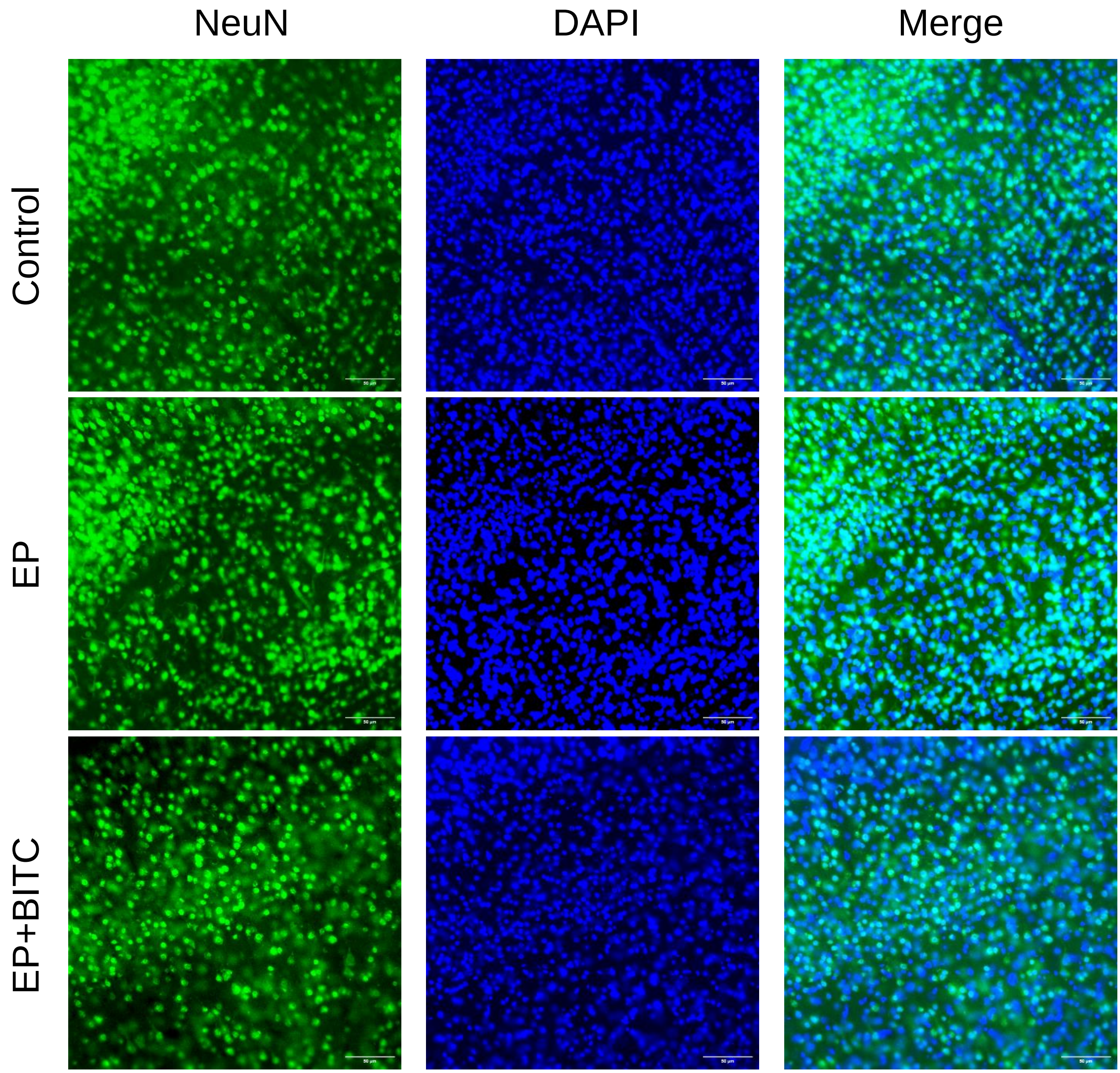

B

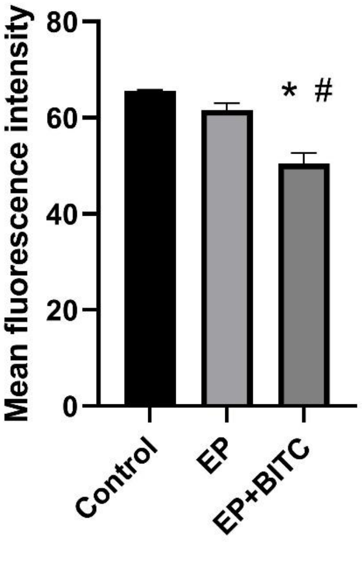

Fig. S4. Immunofluorescence staining of NeuN in cortex region. (n=3 per group; mean ± S.E.M.). (B) Mean fluorescence intensity in the cortex region in the three groups of mice. * Compared with control group: $P < 0.05$, # compared with EP group: $P < 0.05$. One-way ANOVA followed by post hoc least significant difference [LSD] multiple comparison tests.
